# Autonomous Tumor Signature Extraction Applied to Spatially Registered Bi-Parametric MRI to Predict Prostate Tumor Aggressiveness: A Pilot Study

**DOI:** 10.3390/cancers16101822

**Published:** 2024-05-10

**Authors:** Rulon Mayer, Baris Turkbey, Charles B. Simone

**Affiliations:** 1 Department of Radiation Oncology, University of Pennsylvania, Philadelphia, PA 19104, USA; 2 OncoScore, Garrett Park, MD 20896, USA; 3 Molecular Imaging Branch, National Cancer Institute, National Institutes of Health, Bethesda, MD 20892, USA; ismail.turkbey@nih.gov; 4 New York Proton Center, New York, NY 10035, USA; csimone@nyproton.com

**Keywords:** logistic probability, prostate cancer, bi-parametric magnetic resonance imaging (BP-MRI), Gleason score (GS), signal to clutter ratio (SCR), regularization, principal components filtering, clinically significant prostate cancer, correlation coefficient

## Abstract

**Simple Summary:**

The proper management of prostate cancer requires accurate assessment of patients diagnosed with prostate cancer, a common and often lethal cancer. Current conventional prostate cancer diagnosis relies on: (1) needle biopsy, which can result in side effects such as hemorrhage or infection, as well as misplacement of needles resulting in evaluation inaccuracy, (2) prostate serum antigen detection that can lead to overdiagnosis, or (3) MRI interpretations using the Prostate Imaging-Reporting and Data System (PI-RADS), which is a subjective method that depends on the experience of the radiologist’s interpretation of the images. Quantitative analysis of MRI can alleviate the limitations of conventional approaches. Artificial intelligence applied to MRI requires big training datasets, and its application is restricted to patients scanned under certain restricted conditions. More recently, spectral/statistical techniques, adapted from remote sensing, have been applied and tested in spatially registered multi-parametric MRI. The new spectral/statistical techniques require limited training, are flexible regarding patient scanning conditions, and perform well relative to other techniques. This study extends spectral/statistical techniques to make them more independent of the individual radiologist by autonomously finding the appropriate tumor signature within a prostate and, therefore, providing an objective, non-invasive, accurate means for determining prostate tumor aggressiveness.

**Abstract:**

Background: Accurate, reliable, non-invasive assessment of patients diagnosed with prostate cancer is essential for proper disease management. Quantitative assessment of multi-parametric MRI, such as through artificial intelligence or spectral/statistical approaches, can provide a non-invasive objective determination of the prostate tumor aggressiveness without side effects or potential poor sampling from needle biopsy or overdiagnosis from prostate serum antigen measurements. To simplify and expedite prostate tumor evaluation, this study examined the efficacy of autonomously extracting tumor spectral signatures for spectral/statistical algorithms for spatially registered bi-parametric MRI. Methods: Spatially registered hypercubes were digitally constructed by resizing, translating, and cropping from the image sequences (Apparent Diffusion Coefficient (ADC), High B-value, T2) from 42 consecutive patients in the bi-parametric MRI PI-CAI dataset. Prostate cancer blobs exceeded a threshold applied to the registered set from normalizing the registered set into an image that maximizes High B-value, but minimizes the ADC and T2 images, appearing “green” in the color composite. Clinically significant blobs were selected based on size, average normalized green value, sliding window statistics within a blob, and position within the hypercube. The center of mass and maximized sliding window statistics within the blobs identified voxels associated with tumor signatures. We used correlation coefficients (R) and *p*-values, to evaluate the linear regression fits of the z-score and SCR (with processed covariance matrix) to tumor aggressiveness, as well as Area Under the Curves (AUC) for Receiver Operator Curves (ROC) from logistic probability fits to clinically significant prostate cancer. Results: The highest R (R > 0.45), AUC (>0.90), and lowest *p*-values (<0.01) were achieved using z-score and modified registration applied to the covariance matrix and tumor signatures selected from the “greenest” parts from the selected blob. Conclusions: The first autonomous tumor signature applied to spatially registered bi-parametric MRI shows promise for determining prostate tumor aggressiveness.

## 1. Introduction

The accurate assessment of patients diagnosed with prostate cancer, a common and potentially lethal cancer [1], is essential for effective management of the disease [2,3,4,5]. Some conventional approaches such as prostate serum antigen measurement result in overdiagnosis of clinically insignificant tumors, resulting in excessive treatment [6,7,8]. Another approach, needle biopsy, can easily under-sample the tumor due to needle misplacement and can possibly lead to side effects such as infections or hemorrhage [9,10,11,12]. To alleviate under-sampling and side-effect issues, MRI [13,14] and multi-parametric MRI (MP-MRI) [15,16,17] have been employed more recently to non-invasively assess prostate cancer. To reduce patient discomfort, possible side effects and increase patient flow through the clinic, bi-parametric MRI—unlike multi-parametric MRI—does not require injection of contrast material and has been investigated as a simpler alternative diagnostic approach [18,19,20,21]. Currently, MRI studies are qualitatively evaluated using the PI-RADS protocol [22] which relies on visual inspection of the MRI by the radiologist and can result in variable interpretation among radiologists [23]. To further elevate prostate cancer evaluation, quantitative approaches such that apply artificial intelligence (AI) algorithms [24,25,26,27] to MRI aim to provide accurate, reliable, and objective prostate cancer assessments and ameliorate the deficiencies in other approaches. AI algorithms often emulate neuronal processes in combining features to characterize tumors, unlike the present study that uses an analytic approach employing a single equation. The artificial intelligence (AI) algorithms may or may not use the spectral aspect of multi-parametric MRI (unlike this study which only uses the spectral features of the spatially-registered dataset). The artificial intelligence approaches often rely on large sets of training data that use expert annotations from radiologists delineating prostate tumors on the MRI. Such approaches process and evaluate the entire tumor, not the individual voxels within the tumor such as in this study. 

More recently, spectral/statistical approaches have been adapted from remote sensing and applied to predict prostate tumor aggressiveness in multi-parametric [28,29,30,31] and bi-parametric MRI [32]. The individual sequences must be registered to the voxel-level. A vector is associated with each spatially-registered voxel composed of the values in the individual sequence. A tumor is characterized as a signature vector that is distinguished from the average normal prostate signature vector due to the physiological differences such as tissue density and proton diffusion. The background (normal tissue) is further characterized by a multispectral covariance that helps avoid issues of correlation among the sequences when combining them in the calculations through “dewhitening”. Furthermore, noise reduction and departures from statistical normality can be corrected by filtering out noisy principal components and applying regulation to the covariance matrix. Unlike artificial intelligence approaches, the training sets are minimal.

The current implementation of the spectral/statistical approach requires a clinician to identify tumor like voxels within the spatially registered MRI hypercube [28,29,30,31,32]. A color composite is used to quickly identify candidate target voxels through the appearance of a specific color. For example, for bi-parametric MRI, red, green, and blue colors can be assigned to the Apparent Diffusion Coefficient (ADC), the High B-value (HBV), and T2 channels, respectively. In this case, the tumor appears as green due to its relatively high density and low diffusion (low ADC and low red; low diffusion, large HBV and large green; and high density, low T2 and low blue). Clinical intervention can introduce more variation due to the dependence on the clinician’s experience and training, thereby complicating studies and potentially diminishing clinical assessment. The clinician intervention can also slow the evaluation process and reduce the workflow and efficiency into the clinic. In addition, analyzing large number of patients (>1000) for research studies requires greater efficiency in processing and can be abetted by automating the tumor signature extraction process.

This manuscript describes and assesses a novel technique. This investigation is the first and only one to autonomously extract vector target signatures that characterize the tumor without the manual intervention of a clinician or image analyst as in previous spatially registered MP- and BP- MRI studies [28,29,30,31,32]. Unlike most studies, this investigation employs a spectral/statistical algorithm via an analytical approach requiring few parameters and limited training, which processes each voxel within the prostate and tumor. Specifically, this manuscript summarizes how well these signatures perform when inserted into spectral algorithms in terms of accurately finding tumor aggressiveness and clinical significance. Various options for processing the covariance matrix, a mathematical entity that characterizes the normal prostate clutter background, are evaluated in terms of achieving maximal accuracy.

## 2. Materials and Methods

### 2.1. Overview

**Figure 1:**
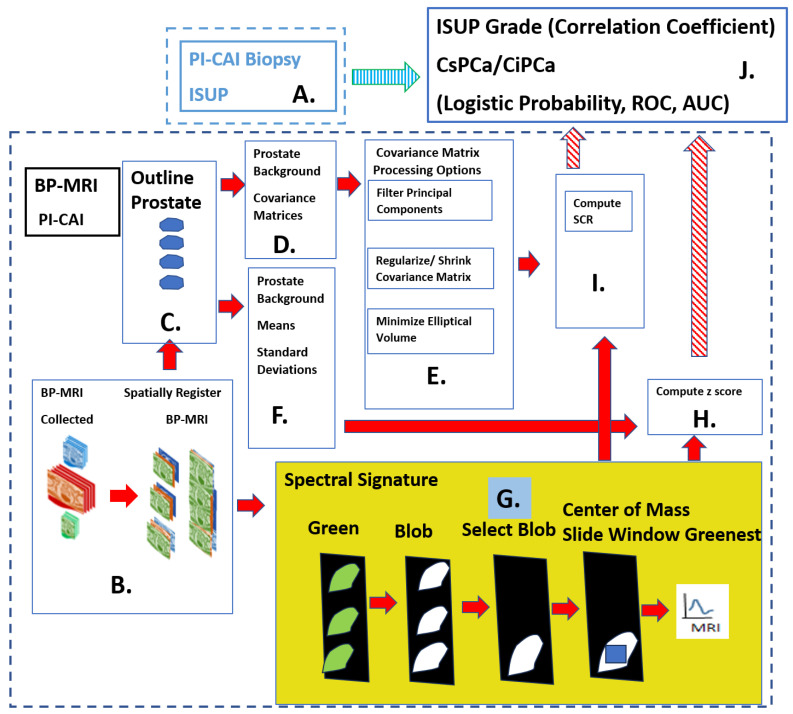
Overall schematic depicting the processes needed to predict the ISUP and CsCPa. Histopathology provides the “Gold standard” ISUP, CsPCa (Panel A). Spatially registered bi-parametric MRI hypercubes are digitally assembled (Panel B). Normal prostate is digitally outlined (Panel C). Background, normal prostate covariance matrix computed (Panel D), Covariance matrix treatments for Signal to Clutter calculations (Panel E). Additional simple background statistics computed (Panel F) Autonomous target signature is depicted in the yellow highlighted panel, the focus of this research (Panel G). z-score computed using statistics and signature from Panels F, G (Panel H), SCR computed using processed covariance and signatures from Panels E, G (Panel I), Predictions of ISUP, CsPCa using z-score or SCR from Panels I,H (Panel J). Fitting between ISUP, CSPCA and Predictions from Panels A,J.

Figure 1 shows the overall scheme to predict the International Society of Urological Pathology (ISUP) grade [33]. The ISUP is the “ground truth” for this study, independently determined from pathology and is related to the Gleason score, a measure of a tumor’s aggressiveness, and Clinically Significant (Insignificant) Prostate Cancer or CsPCa (CiPCa). The prediction of ISUP proceeds in certain sequential steps. Two spectral features, namely z-score (Panel H) and processed Signal to Clutter Ratio (SCR) [31,32] (Panel I) are generated from spatially registered MRI and are different and competing (in this study) quantitative indicators of tumor aggressiveness. For this study, patient MRI data were gathered as part of the PI-CAI Grand Challenge [34]. The ISUP grade is determined from the pathology analysis recorded in the PI-CAI data collection [35] (Panel A in Figure 1). Using the BP-MRI data [28,29,30,31,32], spatially registered hypercubes were assembled from the individual MRI sequences through digital resizing, translation and cropping, specifically the ADC, HBV, and T2 (Panel B in Figure 1). The prostate was manually outlined to create a digital mask (Panel C in Figure 1). The background or normal prostate statistics are characterized through computing the covariance matrix (Panel D) and the mean vector and standard deviation for each MRI channel (Panel F). Various options exist to reduce noise and ensure proper normality through covariance matrix processing (Panel E) for input to SCR (Panel I). Specifically, principal component filtering, regularizing the covariance matrix and elliptical volume minimization reduced the noise (Panel E) in the SCR [31,32] (Panel I). The novel component of this research was that tumor signatures (see yellow filled box and Panel G in Figure 1) were generated autonomously, not manually, to provide input for the z-score and SCR computation [28,29,30,31,32]. The yellow filled panel (Panel G) illustrates the autonomous signature formation process. Details are discussed in the following subsections of the Section 2. First, the autonomous tumor signature generation creates a “normalized quantitative green” image by combining normalized sequences of High B/(ADC+High B+T2). Next, procedures employ thresholding to the quantitative green image, applying morphology operations such as blobbing and removing small blobs. The optimal blob was selected based on blob and sliding window statistics. The center of mass and “greenest” sliding windows were used to select a specific voxel within the blob for the tumor signature. The autonomous signature provides input for features Signal to Clutter Ratio (SCR), (Panel I) and z-score (Panel H). The processed SCR (Panel I) and z-score (Panel H) were linearly (logistical probability) fitted to the ISUP grade (CsPCa/CiPCa) (Panel J) and the PI_CAI biopsy (Panel A), respectively. Quantitatively evaluation of the linear and logistic fits was achieved by computing the correlation coefficients (R) and the Area Under the Curve (AUC) from Receiver Operator Characteristic (ROC) [28,29,30,31,32], respectively (Panel J).

Algorithms to spatially register the bi-parametric data, image process, and compute the SCR and AUC from the ROC curve were implemented using the Python 3 programming language.

### 2.2. Study Design and Population

The patient data were derived from the PI-CAI [34] which is a publicly available repository for prostate tumor MRI and assessments. An annotated multi-center, multi-vendor dataset of 1500 bp MRI exams featuring basic clinical and acquisition variables compose the PI-CAI dataset [34]. Only a subset of the 1500-patient cohort underwent or had available biopsy results. Patients were scanned at a number of different centers by Siemens and Philips scanners. The PI-CAI data collection [34] only comprises bi-parametric MRI, namely ADC, HBV, and T2 sequences.

All patients in this study cohort from the PI-CAI database [34] had biopsy-proven adenocarcinoma of the prostate. This study included 42 consecutive biopsied patients. The mean patient age was 65.1 years (range, 50 to 78 years), the mean PSA was 13.49 ng/mL (range, 1.5 to 81.95 ng/mL), the mean prostate volume mean was 60.6 cm^3^ (range, 19 to 192 cm^3^), and mean ISUP grade was 1.12 (range, 0 to 5). All cases had been anonymized prior to analysis.

### 2.3. Spatial Registered Hypercube Assembly: Magnetic Resonance Imaging

The bi-parametric MRI data [34] was restricted to ADC, HBV, and T2 sequences. Diffusion-weighted MRI sequences were computed using a number (typically five) of increasing magnetic field gradients. In higher magnetic field gradients (HBV), the higher diffusing protons (not tumor) more readily attenuate their signals, producing lower values and were hypointense. On the other hand, tumor nuclei (slower diffusing) under higher magnetic field gradient (HBV) signals are less attenuated and yielded relatively larger values and were hyperintense. The ADC was a slope from a linear fit to the magnetic field gradient images (typically five) for each voxel. Slower diffusing nuclei (tumors) yielded a smaller slope, lower ADC values, and therefore were hypointense. The ADC display higher noise levels relative to each of the images from the magnetic field gradients resulting from a fit. Tumors have higher tissue density and therefore resulted in relatively smaller T2 signals.

### 2.4. Spatial Registered Hypercube Assembly: Image Processing, Pre-Analysis

To initiate spatial registration, the inter-voxel distance and spatial offsets for each of the MRI sequences (ADC, HBV, and T2) were read from information stored in the dicom header files. The MRI images were digitally resized [28,29,30,31,32] to the MRI sequence with the lowest transverse spatial resolution (ADC, HBV). Based on the offsets listed in the image header files, the images were translated a few voxels to the reference image (ADC, HBV). The slices were axially translated to match the offsets using the known location of the axial offsets. Small transverse translation adjustments were applied to the T2 image to match the ADC, HBV from visual inspection. Stacked individual slices that had been scaled, translated, and cropped based on the described protocol are known as a “cube”. The components (ADC, HBV, T2) of the “cube” were spatially registered at the voxel level. The “cubes” associated with each axial slice were then “stitched” together into a narrow three-dimensional hypercube, in order to depict the entire body within the MRI scan Field of View (FOV). These “three-dimensional” (two transverse directions plus spectral dimension composed of ADC, HBV, and T2 images) thereby depicts a four dimensional “object” (three-dimensional body plus the spectral dimension). Such a construct helps increase the processing speed and analysis. For each patient, spatial registration and processing took a few seconds on a Windows 10, base speed 2 Ghz, cache memory 8 GB machine.

**Figure 2:**
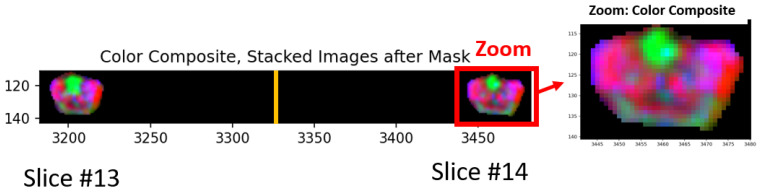
Section of color composite hypercubes where red, green, and blue were assigned to ADC, High B-value, and T2, respectively. Only small sections of slices #13, #14 (out of 27) are shown. The numbers listed along the vertical and horizontal edges show the row and columns numbers, respectively, for the hypercube. The yellow vertical line marks the divider. Red outlined rectangle denotes zoomed region. Zoomed region shown. Note the green region in central, anterior transition zone and dark green in posterior extending from left to right peripheral zone.

Figure 2 shows a color composite derived from the three (ADC, HBV, T2) spatially registered images. Spatially registered cubes derived from each slice are “stitched” together and placed side by side in the horizontal display. In Figure 2, 27 slices of 256 × 256 × 3 cubes (rows × columns × dimensions) were stitched into a single hypercube of size 256 × 6912 × 3 (rows × columns × dimensions). Only small sections of slices #13, #14 (out of 27) are shown. The numbers listed along the vertical and horizontal edges show the row and columns numbers for the hypercube, respectively. The yellow vertical line marks the divider (at column 3328 = 13 × 256) between #13, #14 slides. Only the normal prostate and prostate tumors appear in Figure 2 and all other tissues, bones, etc., in the MRI field of view are blackened out due to masking. Color composite is shown by assigning red, green, and blue to ADC, HBV, and T2, respectively. For greater clarity for viewing the tissues, a zoomed-in portion of the image accompanies the composite color image. The tumor appears as green in such a color scheme (low ADC, high HBV, and low T2).

### 2.5. Normalized “Green” Image

Prostate tumors generally have higher cellular density relative to a normal prostate and that tends to inhibit the diffusion of protons with resultant restricted diffusion rates [4]. In multi- and bi-parametric MRI prostate cancer imaging, tumors appear to show lower, higher and lower signal intensity relative to normal tissue in ADC, High B-value, and T2 images, respectively. Assigning the red, green, and blue colors to ADC, High B-value and T2 means that tumors appear as green. The assignment of the red, green, blue channels was selected arbitrarily, but this study required a standard assignment. Quantifying the “greenness” of a voxel helps detect the blobs related to tumors within a prostate and also sort the blobs based on tumor aggressiveness indicator.

This study generated a normalized green image by adhering to the following procedure [29]. After the bi-parametric MRI was spatially registered, a normal prostate mask marked the normal prostate. From visual inspection, the distributions of ADC, HBV, T2 follow a roughly normal distribution. For voxels residing within the prostate (by using the mask), a cumulative histogram for prostate voxel values in each sequence (ADC, High B-value, T2) determined the 1 percentile and 98 percentile values. The voxels were scaled or stretched based on the 1% and 98% values and anomalous voxels did not distort the normalization. However, aberrant voxels (<1% and >98%) were assigned 0 and 1 values, respectively. The “green” image was then formed by combining the normalized sequences through the ratio: Green/(Red+Green+Blue) or High B/(ADC+High B+T2). The most “tumor-like” voxels presumably possessed the largest green values.

**Figure 3:**
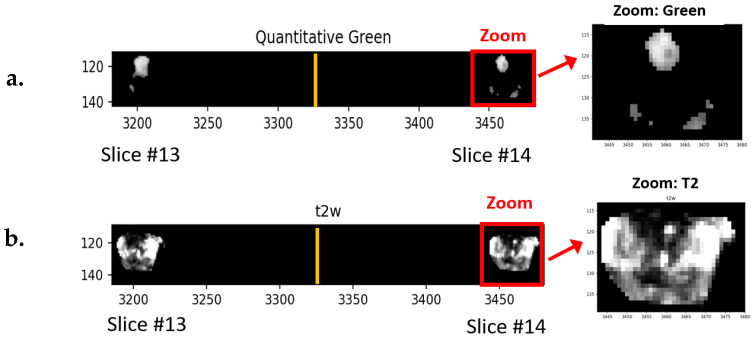
(**a**) Section of normalized green image. Highest green regions are grey, white and no-green is black. Only small sections of slices #13, #14 (out of 27) are shown. The numbers listed along the vertical and horizontal edges show the row and columns numbers, respectively, for the hypercube. The yellow vertical line marks the divider zoomed region shown inside the red rectangle and expanded on right. (**b**) Section and zoomed T2 image. Note hyperintensity in (**a**) “Green” corresponds to hypointense region in (**b**) T2.

Figure 3a shows an example of a stitched quantitative green image. As in Figure 2, only a small portion (slices #13, #14) of the hypercube (27 slices) is shown. In addition, masking only permits display for the prostate and tumor. The white and grey regions show voxels having values exceeding the threshold and are potential blobs and tumors. Note the regions are in the center anterior regions and the left and right posterior regions of the prostate. Zoomed regions are denoted by the red rectangle. For reference, Figure 3b shows the corresponding T2 image. Note the hyperintense region in the Figure 3a “Green” region corresponds to the hypointense region for Figure 3b T2 image.

### 2.6. “Green” Blob Formation

This study generates and examines agglomerations of voxels that presumably depict prostate tumors [31]. The procedure for generating blobs follows a fixed sequence applied to all patient data. First, a threshold (=0.90) is applied the normalized “green” image and voxels exceeding the threshold were assigned a binary value 1 (or True), and those below the threshold were assigned a binary value 0 (or False). The True voxels are only connected if they have a True neighbor voxel residing within a single voxel in the horizontal, vertical, diagonal directions. To eliminate excessively unusual shapes, such as thin string appendages, morphology operations are applied to the blobs, such as erosion being applied to non-zero voxels within structure element (2, 2) to shrink the object and followed by dilation of the remaining non-zero voxels within the structure element (3, 3) to expand the remaining object. Blob sizes less than 12 voxels or roughly 0.1 cc were filtered out. 

**Figure 4:**
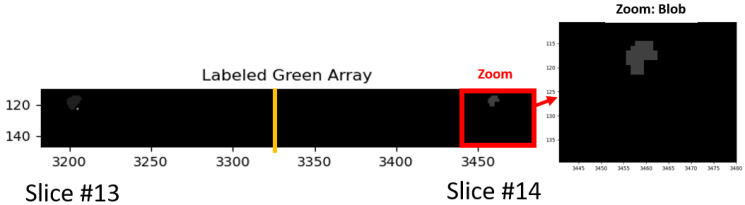
Following thresholding of the normalized green region, the voxels exceeding the threshold were eroded, dilated, and small blobs were filtered out. Zoom region denoted inside red outline rectangle and expanded on right.

Figure 4 shows an example of a “blobbed” selection, labeled after morphology operations erosion, dilation, and filtering. As in Figure 2, only a small portion (slices #13, #14) of the hypercube (27 slices) are shown. In addition, masking only permits display for the prostate and tumor. The red rectangle denotes the zoomed region. Note that after erosion and dilation, the posterior regions were filtered out. 

### 2.7. Blob Selection

Tumors are often multifocal and occupy spatially distinct regions within a given patient with prostate cancer. This study characterizes each blob and computes the average grade over all blobs. In addition, this study examined the selection of a single blob and its relationship with the pathologist’s report on the ISUP grade and clinical significance. The blobs were selected from the normalized “green” image. The possible criteria for selecting a given blob are maximal blob size, maximal “green”, minimal standard deviation, maximal sliding window “green”, minimal sliding window stand deviation, maximal sliding window covariance (average green/standard deviation), and relative blob position in the scan. All blobs for a given patient were rated relatively, based on these statistical and sliding window values. Some of these criteria play a more critical role in predicting tumor aggressiveness. From trial and error, it was found that the following weights helped predict ISUP and clinical significance: Size = 1, Green = 2, Sliding Window Maximum = 3, Sliding Window Covariance = 3, Standard Deviation = 1, Blob Eccentricity = 0, Blob Position = 1, Blob Covariance = 2).

### 2.8. Sliding Windows

**Figure 5:**
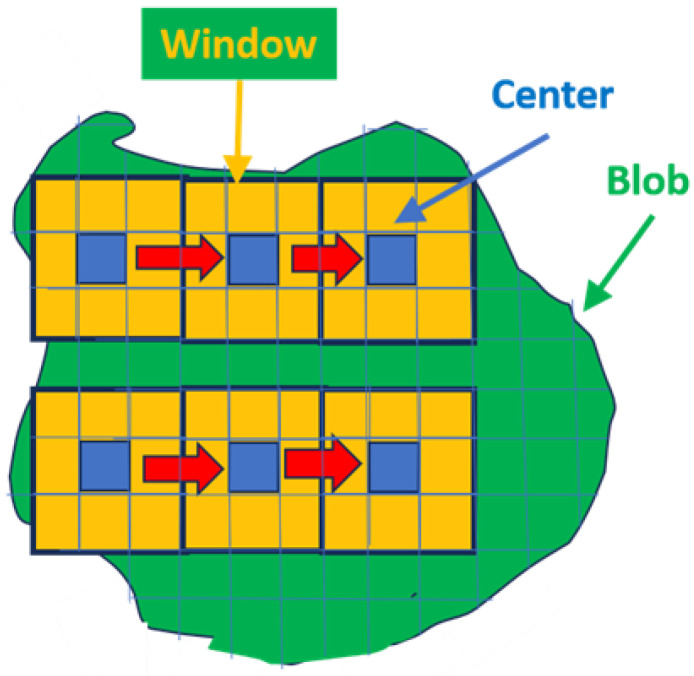
Schematic illustrating the sliding window procedure. Blue denotes box center, yellow denotes surrounding voxels within box, red arrow denotes movement of sliding window.

Tumors are heterogenous, which complicates the autonomous search for the appropriate voxel within the blob that accurately characterizes the tumor. A “sliding windows” approach, shown schematically in Figure 5, helps one find the appropriate voxel within a given blob. Specifically, sliding windows (shown in yellow and blue in Figure 5) find a high “green value” but are also relatively homogenous within a heterogeneous blob (shown as green in Figure 5). In this study, the sliding window is defined as nine voxels (yellow and blue in Figure 5) that include and surround a central voxel (blue in Figure 5). Average and standard deviations are computed for the nine-voxel window and stored. The next window center is translated one voxel away, and the statistics are calculated and stored. This procedure is replicated and extended over the entire blob. The sliding window recording the highest “green” and highest covariance (average/standard deviation) is noted. The “greenest” voxel is based on the highest recorded covariance value from the sliding window cohort. 

### 2.9. Overall Quantitative Metrics Description: SCR, Z-Score

Following common clinical practice, trained radiologists qualitatively determine a tumor’s aggressiveness through visual inspection of multiple MRI pulse sequences [2]. In this study, by contrast, a tumor’s departure from normal prostate tissue was quantitatively expressed through computation of the SCR and z-score (Appendix A.1). The z-score and SCR formulation merge information derived from all MRI sequences. SCR and z-score compute the difference between the mean tumor signature value and the mean normal prostate value, scaled by the normal prostate standard deviation for each MRI sequence (ADC, HBV, and T2). However, the z-score does not account for correlation among the MRI sequences (ADC, HBV, and T2). The SCR, in contrast, decorrelates the sequences via whitening. However, the SCR adds noise. The SCR computation requires generating the covariance matrix. Using the covariance matrix in the SCR calculation decorrelates the different components (i.e., especially the correlation between ADC and DWI) to generate the true contribution of each sequence. The Appendix A.1,Appendix A.2,Appendix A.3 summarize the mathematics behind the SCR algorithm. See references [31,32] for more details.

For each patient, SCR calculations took a few seconds to process on a Windows 10, base speed 2 Ghz, cache memory 8 GB machine.

### 2.10. SCR: Filtering Noise

The covariance matrix in the SCR computation can be decomposed into principal components [36]. Eigenvectors or principal components linearly combine all MRI components. The principal components are all mutually orthogonal to each other and therefore are decorrelated from each other. Conventionally, the ordering of the principal components follows their associated eigenvalue (or statistical variation). Images associated with high eigenvalues have large variations within the image, resulting in better-resolved images. In contrast, images associated with principal components with small eigenvalues are noisy. Filtering out and eliminating the noisy (low eigenvalue) principal components reduces the noise in the covariance matrix calculation and increases the SCR calculation accuracy. The Appendix A.2 summarizes the mathematics for filtering principal components. See references [31,37,38] for more details.

### 2.11. Regularization and Shrinkage

Regularization ensures that the computations using the corrected covariance matrix more closely follow the normal distribution. The analytic equation describing the covariance matrix only results in an approximation to what would be expected for normality. Shrinkage regularization [31,32,39] perturbs the original covariance matrix CM(γ) by adding a diagonal matrix weighted with a mixing parameter γ to generate a regularized or modified regularized covariance matrix. Through minimizing, the discriminant function helps to choose the appropriate γ. Regularized and modified regularized covariance matrix computations use the same procedure but only differ in the different choice of the mixing diagonal matrix (identity vs. diagonal of standard deviations). The Appendix A.3 describes a summary of the mathematics behind regularization procedures. See references [31,32,39] for more details.

**Figure 6:**
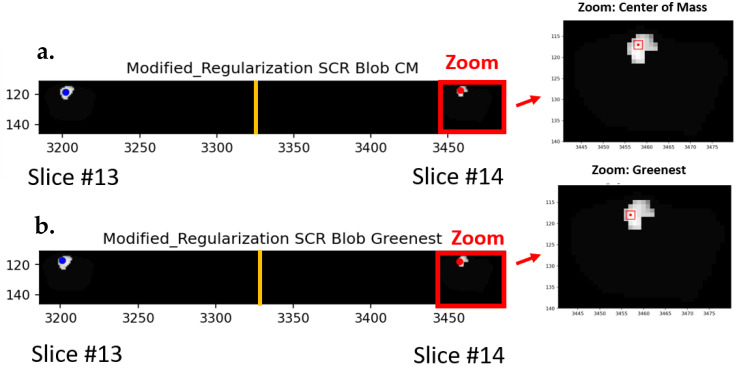
The (**a**) center of mass (CM) and the (**b**) “greenest” sliding window voxel are also shown for SCR generated from covariance matrix treated with modified regularization. Red rectangle shows the zoomed in region. Red square shows regions of analysis within the blob.

Figure 6 shows the center of mass (Figure 6a), and the “greenest” sliding window voxel (Figure 6b) are also shown for SCR generated from covariance matrix treated with modified regularization. As in Figure 2, only a small portion (slices #13, #14) of the hypercube (27 slices) is shown. In addition, masking only permits display for the prostate and tumor. The red rectangle shows the zoomed region. The specific windows are denoted as red squares showing the region of analysis within the blob.

### 2.12. Elliptical Volume Minimization

Another way to reduce noise in the covariance matrix calculation is to apply Elliptical Volume Minimization (EVM) [40]. For this study, EVM sequentially removes 10% of some randomly chosen pixels, then searches and computes the elliptical hypervolume for the remaining 90% of the prostate voxels. The location of the 90% remaining voxels and their elliptical hypervolume for each randomly chosen sequence is recorded. The minimum elliptical volume is chosen, presumably reducing the effects of the 10% aberrant voxels.

### 2.13. Logistic Regression

Logistic regression [41] fit the processed SCR, z-score, or patient data to the dependent categorical variable, CsPCa, for this study. The ISUP grade is derived from the pathological assessment from analysis of the MRI biopsy, systematic biopsy, the combination of MRI and systematic biopsy or histopathology of the radical prostatectomy. The clinically significant PCa (CsPCa) was assigned to ISUP grade ≥ 2, and the clinically insignificant PCa (CiPCa) was assigned to <2. The training/test sets were randomly assigned among the 42 patients but maintained a 70%/30% ratio for all combinations of patient sets. This study generated 1000 new randomized sets or configurations of results of patients within the training/test conditions. These configurations resulted in 1000 independent ROC [42] curves, resulting in a distribution of AUC values. The distribution of AUC scores was recorded, and the 2.5% and 97.5% largest AUC delineated the 95% confidence interval. The fit quality was assessed through the AUC and the 95% confidence interval in the AUC distribution.

## 3. Results

**Figure 7:**
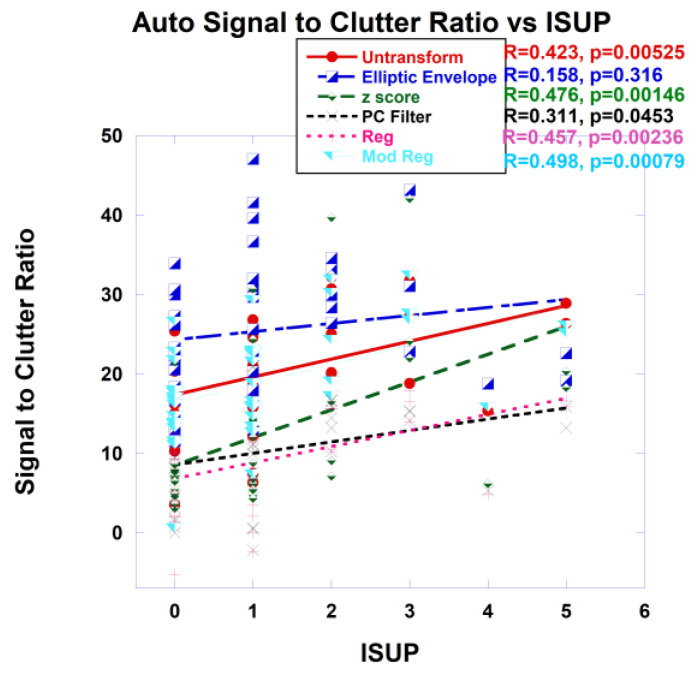
Signal to Clutter Ratio plotted against ISUP for the “greenest” voxel of selected blobs. For SCR fitting, covariance matrix was untransformed, elliptical envelope applied, PC filtered, and regularized and modified regularized. Fitting using z-score also shown. Correlation coefficients and *p*-values shown for each covariance treatment.

Purported tumor signatures were found among the blobs and inserted into the whitened Euclidean distance, or SCR computation and plotted against the ISUP. One option is to find the average target signature taken from all blobs. In addition, blobs that are more likely to depict a tumor’s aggressiveness were selected based on the blob’s statistics (average, standard deviation), statistics from the sliding windows within each blob, size and position within the scan. Within each blob, there was the option of choosing a signature from the center of the mass or from the “greenest” voxels as determined by the sliding window. As an example, Figure 7 shows plots of SCR from autonomous signatures gathered from the “greenest” part of the selected blob. The covariance matrix was untransformed, approximated with the z-score, elliptical envelope, PC filtered, regularized and modified regularized. The combination of z-score and modified regularization results in the highest (lowest) correlation (*p*-value).

Table 1 shows the metrics, specifically the correlation coefficient and p-values for assessing the fitting of SCR with ISUP for tumor signatures taken from averaging the signature from all blobs using the center of mass of each blob (Ave Blob CM) or averaging from all blobs using the “greenest” part (Ave Blob Green) of every blob, signature from a selected blob, and the choice of center of mass (Signature CM) or “greenest part of the selected blob (Signature Green). Also shown are evaluations of fits resulting from different manipulations of the covariance matrix for the SCR, such as unprocessed covariance matrix (Untransformed), elliptic envelope (Elliptic), principal component filtering (PC Filter), regularization (Reg), modified regularization (Mod Reg) and from z-score (z-score). The highest (lowest) correlation coefficient (*p*-value) achieved comes from the z-score and modified regularization of the covariance matrix.

**Figure 8:**
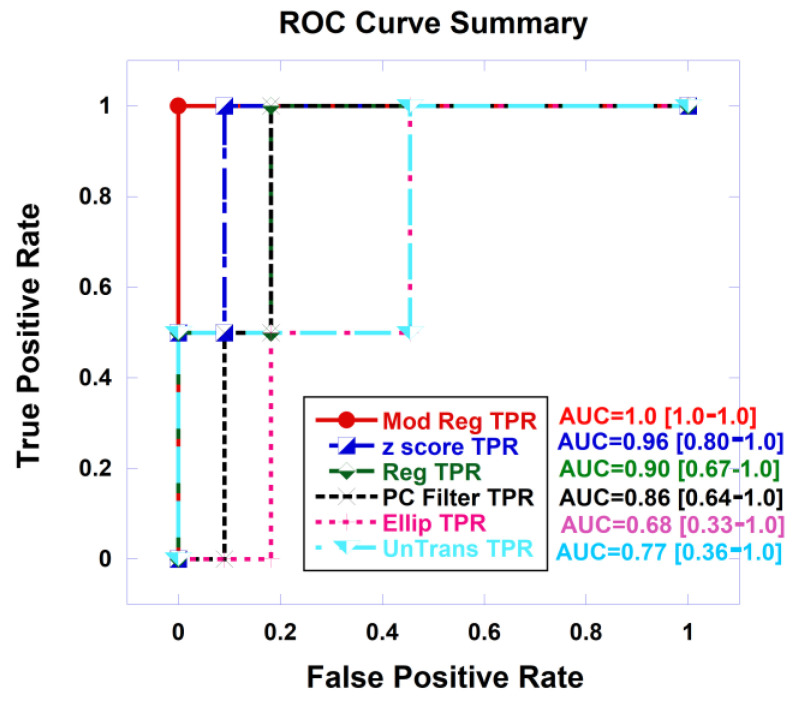
Receiver Operator Curve (ROC) autonomous signatures taken from greenest part of the selected blob, for covariance treatments: untransformed, elliptical envelope, z-score, PC filtered, regularized, modified regularized. AUC and 95% confidence interval also shown.

As an example, Figure 8 shows plots of Receiver Operator Characteristic (ROC) curves that plot the true positive rate and against the false positive rate or false alarm rate from logistic probability fits of the SCR from autonomous signatures gathered from the “greenest” part of the selected blob to the clinically significant prostate cancer. The covariance matrix was untransformed, approximated with the z-score, elliptical envelope was applied, PC filtered, regularized, modified regularized. The combination of z-score and modified regularization results in the highest (lowest) correlation (*p*-value). 

Table 2 shows the metrics, specifically the AUC (along with the 95% confidence interval) from ROC curves, for assessing the logistic probability of fitting SCR with clinical significance for prostate cancer for tumor signatures taken from averaging the signature from all blobs using the center of mass (Ave Blob CM) or the greenest parts of all blobs (Ave Blob Green), signature from a selected blob, and the choice of center of mass (Signature CM) or “greenest part of the selected blob” (Signature Green). The highest AUC achieved comes from the z-score and modified regularization of the covariance matrix.

## 4. Discussion

This study discusses the procedures for autonomously generating prostate cancer signatures and the analysis for how well these signatures perform when inserted into spectral algorithms in terms of accurately finding tumor aggressiveness and determining clinical significance. This study autonomously finds regions of tumors or blobs, autonomously searches within the blobs for an appropriate voxel that depicts the patient’s tumor, and uses the extracted signature associated with the selected voxel to help determine the tumor’s aggressiveness. This study extracts signatures through purely spectral, not spatial processing and does not employ training data sets. This study finds that achieving high correlation coefficients, low *p*-values (< 0.01) for fitting to ISUP and high AUC (> 0.90) from logistic probability fits to clinically significant prostate cancer is achieved if the covariance matrix is approximated with z-score and processed using the modified regularization approach. Greater accuracy was achieved by selecting tumor blobs based on statistics applied to sliding windows, choosing the “greenest” sliding window. A reduction in accuracy resulted from averaging signatures from all blobs.

Previously, prostate tumors were identified and selected manually by the clinician [28,29,30,31,32]. Autonomous tumor signature presumably is more objective and not subject to training and experience of the radiologist. A more objective approach would presumably aid research protocols by reducing the variability in patient assessments. Furthermore, autonomous target signature selection should expedite the patient assessment and increase clinical efficiency and patient throughput. 

Despite the high correlation, low *p*-values, and high AUC values found from the autonomous search, tumor signatures derived from human intervention performed even better [32]. Future work should focus on selecting better candidate blobs. Choosing the signature derived from averaging over all blobs performed well, but not as well as selecting a particular blob based on size and statistical metrics. The selection criteria used in this study, specifically statistics of the blob (size, blob statistics, sliding window statistics, position) may not be sufficient in terms of features, weighting of the features. The choice of position within the blob may depend on the size of the blob. In particular, the center of mass choice may be suboptimal for blobs with curvature resulting in misplaced signature position. Possibly a compromise between center of mass and “greenness” is needed when selecting a voxel within the selected blob. Artificial intelligence or rigorous multivariable fitting may find more optimal selection criteria. 

This study found that accurate determination of tumor aggressiveness and clinical significance depends on how the covariance matrix is processed, filtered, regularized. Combining multiple sequences in multi-parametric and bi-parametric MRI must ensure that each sequence is weighted appropriately. Specifically, how sequences can be combined without possibly inappropriately weighting correlated sequences. All corrections removed the mean values of the normal prostate sequences and scaled the differences based on the sequence’s normal prostate standard deviation. This study found using z-score and modified covariance matrix achieved the best performance. Applying the elliptical envelope and not transforming the covariance matrix degraded the accuracy for the tumor aggressiveness.

Tumor structure is complicated and heterogenous. It is composed of vasculature, necrotic tissue, and other structures. Therefore, it can be difficult but essential to determine which specific part of the tumor represents the true indicator of the disease progression. A unique aspect of this study is the use and examination of sliding windows in selecting the specific voxel whose signature accurately helps depict the tumor’s aggressiveness. Following the pathologists’ criteria, this study finds that the signature from the “greenest” sliding window of a selected blob offers the best representation the tumor’s aggressiveness. Future studies may investigate the possibility of biopsy based on the findings of the autonomous tumor signature search. The choice of center of mass selection to select the tumor signature can be confounded by tumors with curvature. Future studies may find combining the “greenest” with the center of mass may provide a better choice. 

In this study, correlation coefficients and p-values from fitting to the ISUP grade and the AUC resulting from logistical fits to the CsPCa/CiPCa achieves comparable values with those from AI [24,25,26,27]. Prospective studies of larger patient populations might confirm the current findings and even potentially demonstrate that spectral/statistical techniques outperform AI. To generate a model, AI requires a substantial amount of training. In addition, AI is only applicable to certain clinical conditions such as a given magnetic field, pulse sequence and AI may need to be retrained for patients scanned under different conditions and scanners. In contrast [28], spectral/statistical techniques is flexible and can manage new clinical situations by applying the “whitening–dewhitening” transform to spectral signatures that require minimal processing.

Due to the use of analytic equations and the absence of fitting parameters, the spectral/statistical algorithms do not follow the familiar training testing, prediction procedures for artificial intelligence. This pilot study examined the feasibility of simplifying the entire process by skipping the conventional training, testing to determine the parameters. With higher patient numbers, a “prediction” phase could be added. Future efforts may use the simple coefficients (offset, slope) from linear regression fitting to ISUP in this study to predict the ISUP or CsPCa of another set of patients.

This study examined and tested a variety of schemes to autonomously find tumors, and search within the blob for appropriate voxels that accurately depict tumors. Prostate tumor signatures extracted from blobs can be inserted into spectral algorithms to determine tumor aggressiveness and the clinical significance of prostate cancer.

Our study has limitations. This study only examined a relatively small, albeit sufficient number of patients to draw statistically meaningful conclusions. This study only applied spectral/statistical approaches to handle the patient data and did not also simultaneously apply an AI algorithm. Direct comparison of spectral/statistical vs. AI approaches applied to the same patient cohort is merited. In addition, this study’s results warrant future investigation with a greater number of patients.

## 5. Conclusions

The first autonomous tumor signature extraction applied to spatially registered bi-parametric MRI shows promise for determining prostate tumor aggressiveness, simplifies the implementation, and reduces burden for patients and clinicians. Further studies such as those using a greater number of patients and better blob selection technique is merited. 

## Figures and Tables

**Table 1:** Correlation coefficient, *p*-values.

Covariance Matrix Manipulation	Correlation Coefficient	*p*-Value
Untransformed Ave Blob CM	0.326	0.0351
Untransformed Ave Blob Green	0.33	0.033
Untransformed Signature CM	0.362	0.0185
Untransformed Signature Green	0.423	0.00525
Elliptic Ave Blob CM	0.219	0.163
Elliptic Ave Blob Green	0.204	0.196
Elliptic Signature CM	0.137	0.388
Elliptic Signature Green	0.158	0.316
Z-score Ave Blob CM	0.45	0.00281
Z-score Ave Blob Green	0.46	0.00218
Z-score Signature CM	0.448	0.00293
Z-score Signature Green	0.476	0.00146
PC Filter Ave Blob CM	0.269	0.0848
PC Filter Ave Blob Green	0.273	0.08
PC Filter Signature CM	0.282	0.0706
PC Filter Signature Green	0.311	0.0453
Reg Ave Blob CM	0.315	0.042
Reg Ave Blob Green	0.321	0.038
Reg Signature CM	0.415	0.00634
Reg Signature Green	0.457	0.00236
Mod Reg Ave Blob CM	0.464	0.00195
Mod Reg Ave Blob Green	0.439	0.0036
Mod Signature Reg CM	0.4473	0.00298
Mod Signature Reg Green	0.498	0.00079

Abbreviations: Untransformed: unprocessed, Elliptic: elliptic envelope, z-score, PC Filter: principal component filtered, Reg: regularized, Mod Reg: modified regularization, CM: center of mass, Ave Blob CM: center of mass from averaging over all blobs, Ave Blob Green: greenest from average over all blobs, Signature CM: center of mass from the selected blob, Signature Green: greenest voxel from selected blob.

**Table 2:** AUC from ROC for logistic probability fit.

Covariance Matrix Manipulation	AUC [95% CI]
Untransformed Ave Blob CM	0.775 [0.500–1.0]
Untransformed Ave Blob Green	0.684 [0.333–1.0]
Untransformed Signature CM	0.681 [0.182–1.0]
Untransformed Signature Green	0.774 [0.364–1.0]
Elliptic Ave Blob CM	0.592 [0.300–0.899]
Elliptic Ave Blob Green	0.592 [0.300–0.899]
Elliptic Signature CM	0.639 [0.333–0.909]
Elliptic Signature Green	0.684 [0.333–1.0]
Z-score Ave Blob CM	0.915 [0.667–1.0]
Z-score Ave Blob Green	0.869 [0.545–1.0]
Z-score Signature CM	0.955 [0.800–1.0]
Z-score Signature Green	0.955 [0.80–1.0]
PC Filter Ave Blob CM	0.911 [0.700–1.0]
PC Filter Ave Blob Green	0.864 [0.583–1.0]
PC Filter Signature CM	0.908 [0.667–1.0]
PC Filter Signature Green	0.865 [0.636–1.0]
Reg Ave Blob CM	0.727 [0.250–1.0]
Reg Ave Blob Green	0.638 [0.083–1.0]
Reg Signature CM	0.864 [0.583–1.0]
Reg Signature Green	0.909 [0.667–1.0]
Mod Reg Ave Blob CM	0.915 [0.667–1.0]
Mod Reg Ave Blob Green	0.869 [0.545–1.0]
Mod Signature Reg CM	0.955 [0.800–1.0]
Mod Signature Reg Green	1.0 [1.0–1.0]

Abbreviations: Untransformed: unprocessed, Elliptic; elliptic envelope, z-score, PC Filter: principal component filtered, Reg: regularized, Mod Reg: modified regularization, CM: center of mass, Ave Blob CM: center of mass from averaging over all blobs, Ave Blob Green: greenest from average over all blobs, Signature CM: center of mass from the selected blob, Signature Green: greenest voxel from selected blob.

## Data Availability

The dataset: https://pi-cai.grand-challenge.org/, accessed on 3 March 2023.

## Appendix A

### Appendix A.1. Overall Quantitative Metrics Description: SCR, Z-Score

The tumor signature component *S_i_* for sequence *i (i* = ADC, HBV, T2) is an average over *W* target values (*x_i,q_*) from the q voxels in the square window (*W* = 9). In this study, autonomous searches select the optimal blob and then apply the sliding windows protocol for finding the center for the target signature by finding the center of mass (CM) or the “greenest” part of the blob for the *x_i,q_*.

(A1) $$S_{i}=\frac{1}{W}\sum_{q=1}^{W}x_{i,q}\;$$

After applying manual delineation of the normal prostate to the spatially registered hypercube by a clinician to determine the normal voxel *x_i,p_*, the mean normal prostate value *μ_i_* for sequence *i* is the average over *N* normal prostate voxels *x_i,p_*.

(A2) $${µ}_{i}=\frac{1}{N}\sum_{p=1}^{N}x_{i,p}\;$$

The *z_score_* [31,32] sums all *M (M* = 3) differences

(A3) $$z_{score}=\frac{1}{M}\sum_{i=1}^{M}{\left(\frac{S_{i}-\mu_{i}}{\sigma_{i}}\right)}^{2}\;$$

that is scaled by the normal prostate standard deviation *σ_i_*, given by

(A4) $$\sigma_{i}=\frac{1}{N}{\left(\sum_{p}^{N}{\left(x_{i,p}-\mu_{i}\right)}^{2}\right)}^{1/2}$$

*z_score_* is simple but does not correct for correlation among the *i* sequences (ADC, HBV, T2).

To account for correlation among the different sequences, the Signal to Clutter Ratio *SCR* [31,32] is given by

(A5) $$SCR={\left(S-\mu \right)}^{T}{CM}^{-1}\left(S-\mu \right)$$

Equation (A5) is a matrix multiplication with three components, namely ADC, HBV, T2. The superscript *T* denotes a vector transpose operation, *CM* is the covariance matrix, and the superscript −1 denotes a matrix inverse operation. *S* is the vector tumor signature (see Equation (A1)). The mean value for normal prostate or background is denoted as *μ* (a vector, see Equation (A2)).

### Appendix A.2. SCR: Filtering Noise, Regularization

Noise in the covariance matrix CM can be reduced by eliminating the noisy contributors, specifically by filtering out the principal components showing the lowest variation. The filtered *SCR_Filtered_* is given by

(A6) $${SCR}_{Filtered}={\left(S-\mu \right)}^{T}{CM}_{Filtered}^{-1}\left(S-\mu \right)\;\;\;\;$$

The inverse covariance matrix *CM_Filtered_*^−1^ *CM*^−1^*_Filtered_* is a square symmetrical matrix and therefore can be decomposed into three parts [36],

(A7) $${CM}_{Filtered}^{-1}=\Lambda^{T}\lambda_{Filtered}^{-1}\Lambda$$

Λ is the eigenmatrix, Λ^T^ is transpose of the eigenmatrix, and diagonal matrix *λ_Filtered_*^−1^ populates the diagonal with eigenvalues *λ*^2^_1_, *λ*^2^_2_, *λ*^2^_3_…*λ*^2^*_M_* [36].

(A8) $$\lambda_{Filtered}^{-1}=\left[\begin{array}{ccccc} \frac{1}{\lambda_{1}^{2}} & 0 & \cdots & 0 & 0\\ 0 & \frac{1}{\lambda_{2}^{2}} & 0 & 0 & 0\\ \vdots & 0 & \ddots & 0 & \vdots \\ 0 & 0 & 0 & 0 & 0\\ 0 & 0 & \cdots & 0 & 0 \end{array}\right]\;\;\;$$

The principal components, eigenvectors, eigenvalues are ordered based on the size of the associated eigenvalue. The eigenvalues range from the largest *λ*_1_ to the smallest *λ_M_*. corresponding to images with high signal and high variation (1, 2) to low variation and noisy *(M* − 1, *M*). Removing or deleting the lowest valued eigenvalues (Mth or 3 in this study) for Equations (A6)–(A8), filters out the noisy eigenvectors [31,32].

### Appendix A.3. Regularization and Shrinkage

Regularization or shrinkage [31,32,39] adds extra components to the covariance matrix *CM(γ)* in order to maximize the normal distribution or equivalently minimize the discriminant function d(γ) [=−ln(normal distribution)]. Specifically, regularization or shrinkage adds a diagonal component that is controlled by the parameter γ and thereby perturbing the covariance matrix *CM* into the regularized *CM_mod___Reg_(γ)*

(A9) $${CM}_{Mod\text{\_}Reg}=\left(1-\gamma \right)CM+\gamma V\;$$

*V* is a diagonal square matrix filled up with the square of the standard deviations from *M* modalities and is given by

(A10) $$V=\left[\begin{array}{ccccc} \sigma_{1}^{2} & 0 & \cdots & 0 & 0\\ 0 & \sigma_{2}^{2} & 0 & 0 & 0\\ \vdots & 0 & \ddots & 0 & \vdots \\ 0 & 0 & 0 & \sigma_{M-1}^{2} & 0\\ 0 & 0 & \cdots & 0 & \sigma_{M}^{2} \end{array}\right]\;\;$$

The covariance matrix *CM*(*γ*) maximizes the normal distribution or equivalently minimizes the discriminant function *d*(*γ*) (=−ln(normal distribution)), by specifically, inserting *CM_Mod_Reg_* (Equation (A9)) and *V* (Equation (A10)) into the modified discriminant function *d_mod_Reg_ (γ)*

(A11) $$d_{Mod\text{\_}Reg}\left(\gamma \right)=\sum_{i=1}^{N}{\left(x_{i}-\mu \right)}^{T}{CM}_{Mod\text{\_}Reg}^{-1}\left(\gamma \right)\left(x_{i}-\mu \right)+\ln(\det({CM}_{Mod\text{\_}Reg}(\gamma )))\;$$

Which is computed for all discreet values of *γ* in the range of 0 < *γ* < 1. A minimum *d_mod_(γ_min_)* located at *γ_min_*_._. This *γmin* is inserted into Equation (A9) resulting in *CM_Mod_Reg_*

(A12) $${CM}_{Mod\text{\_}Reg\text{\_}Min}=\left(1-\gamma_{Min}\right)CM+\gamma_{Min}V\;\;$$

and then subsequently Equation (A12) is inserted into Equation (A5) to form *SCR_Mod_Reg_*

(A13) $${SCR}_{Mod\text{\_}Reg}\left(\gamma =\gamma_{Min}\right)={\left(S-\mu \right)}^{T}{CM}_{Mod\text{\_}Reg\text{\_}Min}^{-1}(\gamma =\gamma_{Min})\left(S-\mu \right)\;\;$$

Another way to regularize the *CM* is by applying a similar, but more standard form of regularization and shrinkage [31,32,39]. Specifically, shrinking the difference in the highest and lowest eigenvalues by adding a diagonal component that is proportional to the identity matrix (with identical values along the diagonal) controlled by the parameter *γ* and thereby perturbing the covariance matrix *CM* into the regularized *CM_Reg_(γ)*

(A14) $${CM}_{Reg}=\left(1-\gamma \right)CM+\frac{Tra(CM)}{M}\gamma I\;\;\;$$

where *Tra* denotes the trace operator, *I* is the identity matrix, and discreet values of *γ* range from *γ* = 0.0 (or no *CM* modification) to *γ* = 1.0 (or *CM* is proportional to the identity matrix). For ordinary regularization, the regularized discriminant function *d_Reg_* (=−ln(normal distribution)) is

(A15) $$d_{Reg}\left(\gamma \right)=\sum_{i=1}^{N}{\left(x_{i}-\mu \right)}^{T}{CM}_{Reg}^{-1}\left(\gamma \right)\left(x_{i}-\mu \right)+\ln(det({CM}_{Reg}(\gamma )))$$

Among the discreet *γ*’s (range 0 < *γ* < 1), the lowest discriminant function *d_Reg_Min_* is found and located at *γ* = *γ_min_* and given by in

(A16) $$\begin{array}{cc} d_{Reg\text{\_}Min}=\sum_{i=1}^{N} & {{(x}_{i}-\mu )}^{T}{CM}_{Reg\text{\_}Min}^{-1}(\gamma =\gamma_{min})\;{(x}_{i}-\mu )+\\ & ln(det{(CM}_{Reg\text{\_}Min}(\gamma =\gamma_{min}\;)))\; \end{array}$$

And again,

(A17) $${CM}_{Reg\text{\_}Min}=\left(1-\gamma_{Reg_{Min}}\right)CM+\frac{Tra(CM)}{M}\gamma_{Reg\text{\_}Min}I\;$$

where the sum is over all *N* samples within the entire prostate marked by the prostate mask, the determinant operation is denoted det. *SCR_Reg_*, or regularized *SCR* is given by

(A18) $${SCR}_{Reg}\left(\gamma =\gamma_{Min}\right)={\left(S-\mu \right)}^{T}{CM}_{Reg\text{\_}Min}^{-1}(\gamma =\gamma_{Min})\left(S-\mu \right)\;$$

Note: *γ* = 0 generates the standard *SCR* (Equation (A5)).
